# Thiamine Deficiency in Tropical Pediatrics: New Insights into a Neglected but Vital Metabolic Challenge

**DOI:** 10.3389/fnut.2016.00016

**Published:** 2016-06-14

**Authors:** Laurent Hiffler, Benjamin Rakotoambinina, Nadia Lafferty, Daniel Martinez Garcia

**Affiliations:** ^1^Dakar Unit, Medical Department, Médecins Sans Frontières (MSF), Dakar, Senegal; ^2^Unit of Nutrition Physiology, University of Antananarivo, Antananarivo, Madagascar; ^3^Pediatric Team, Medical Department, Médecins Sans Frontières (MSF), Barcelona, Spain

**Keywords:** thiamine, thiamine deficiency, tropical pediatrics, beriberi, refeeding syndrome, pediatric critical care, type B lactic acidosis, thiamine transporters

## Abstract

In humans, thiamine is a micronutrient prone to depletion that may result in severe clinical abnormalities. This narrative review summarizes current knowledge on thiamine deficiency (TD) and bridges the gap between pathophysiology and clinical presentation by integrating thiamine metabolism at subcellular level with its function to vital organs. The broad clinical spectrum of TD is outlined, with emphasis on conditions encountered in tropical pediatric practice. In particular, TD is associated with type B lactic acidosis and classic forms of beriberi in children, but it is often unrecognized. Other severe acute conditions are associated with hypermetabolism, inducing a functional TD. The crucial role of thiamine in infant cognitive development is also highlighted in this review, along with analysis of the potential impact of TD in refeeding syndrome during severe acute malnutrition (SAM). This review aims to increase clinical awareness of TD in tropical settings where access to diagnostic tests is poor, and advocates for an early therapeutic thiamine challenge in resource-limited settings. Moreover, it provides evidence for thiamine as treatment in critical conditions requiring metabolic resuscitation, and gives rationale to the consideration of increased thiamine supplementation in therapeutic foods for malnourished children.

## Introduction

Thiamine (Vitamin B1) is an essential micronutrient with dual coenzymatic and non-coenzymatic functions. It is involved in carbohydrate and branched-chain amino acid metabolism, as well as in the production of neurotransmitters, myelin, and nucleic acids. There is also evidence that thiamine plays a role in immune and anti-inflammatory processes and gene regulation (1–4).

As an essential micronutrient, the body’s requirements are exclusively dependent on dietary supply as there is no endogenous synthesis. The combination of limited body storage and a high turnover rate (half-life <10 days) results in potential depletion of thiamine stores within 2 weeks if it is not continuously replaced. In addition, thiamine’s hydrosolubility, coupled with its renal clearance profile, contributes to a propensity to thiamine deficiency (TD) throughout life.

In pediatrics, the overall clinical picture of TD is not easy to recognize, mimicking or being confused with other diseases. Not surprisingly, the likelihood of misdiagnosis of TD is even greater in resource-limited settings (5–7). Despite being easily treatable, TD continues to be seen in all age groups in both high and low resource countries with potentially severe and life-threatening consequences (8–12).

This article focuses initially on thiamine physiology from ingestion in the diet to uptake and metabolism at mitochondrion level and the circumstances leading to TD in tropical pediatrics. By highlighting the essential role of thiamine in various body functions, this article enables us to better understand the spectrum of clinical presentations of TD in children, including the various manifestations of beriberi, refeeding syndrome in malnutrition, severe clinical states requiring resuscitation, and conditions linked to anti-thiamine factors. A brief overview of diagnosis and treatment of TD concludes the paper and gives suggestions for the way forward in the management of TD.

## Thiamine Metabolism: A Physiological Snapshot of Thiamine in Humans

Ingested dietary thiamine is essential to meet body requirements in humans who are unable to synthesize it naturally. The daily requirement of thiamine depends on age, body weight, physiological conditions, and individual metabolism with respect to the overall energy content in the diet.

Unlike for adults, recommendations of the optimal thiamine-caloric ratio (mg/1000 kcal/day of energy consumed) are not yet firmly established in pediatrics (9, 13). In children, whose caloric needs are age dependent, the estimated daily recommended dietary allowance (RDA) is 0.2 mg up to 6 months of age, 0.3 mg from 7 months to 3 years of age, and 0.6 mg between 4 and 8 years of age. Over 8 years, daily RDA varies from 0.9 mg up to 1.2 mg.

The content of thiamine and its derivatives in breast milk is around 0.21 mg/l, but varies widely according to diet and countries. Nevertheless, thiamine-deficient breast milk is not uncommon and can have a serious effect on infant thiamine status during exclusive breastfeeding (14, 15).

### Body Thiamine Distribution

After ingestion, free thiamine absorption occurs mainly in the proximal small intestine either via a saturable carrier-mediated system for low thiamine concentrations, or by passive diffusion for high concentrations. Inside the polarized enterocyte, the vectorial transport of thiamine is through specific human thiamine transporters-hTHTRs (ThTr1 and 2) (16–18), after which free thiamine disperses throughout the cell. Genetic defects in ThTr1 (*SLC19A2*) and ThTr2 (*SLC19A3*) result in thiamine-responsive megaloblastic anemia (TRMA) and biotin-responsive basal ganglia disease (BBGD), respectively (2, 19, 20).

**Figure 1:**
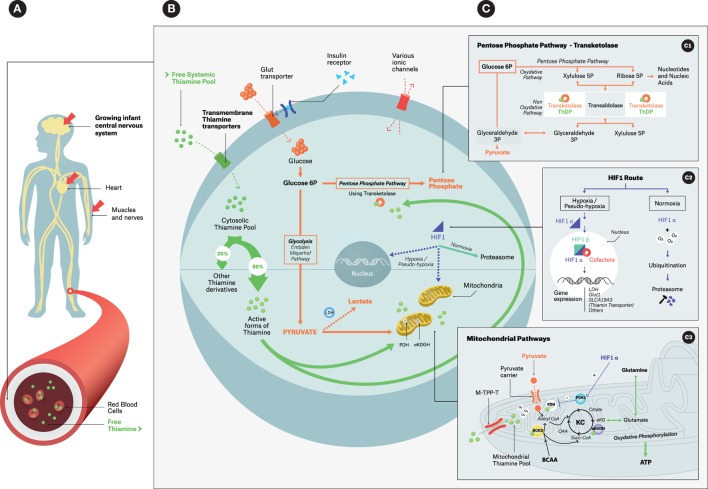
**Simplified overview of thiamine intracellular action: focus on coenzymatic functions**. **(A)** Infant thiamine distribution with its three main target organs; **(B)** thiamine cellular metabolism with its main pools and thiamine-related metabolic pathways; **(C)** zooming boxes: **(C_1_)** – cytosolic pentose phosphate pathways using transketolase, **(C_2_)** – HIF-1 alternative routes, **(C_3_)** – mitochondrial events; ThDP is a cofactor for PDH, α-KGDH and BCKD. Under hypoxia/pseudo-hypoxia HIF1-α upregulates PDK1 that in turn downregulates PDH. Abbreviations: *BCKD*, branched-chain ketoacid dehydrogenase complex; *LDH*, lactic dehydrogenase leading to Type B acidosis; *PDH*, pyruvate dehydrogenase complex; *PDK1*, pyruvate dehydrogenase kinase; α-*KGDH*, alpha-ketoglutarate dehydrogenase. *ATP*, adenosine triphosphate; *BCAA*, branched-chain amino acids; *Glut transporter*, glucose transporter; *HIF*, hypoxia-inducible factor, as a dimeric (α, β unit) transcriptional factor; *KC*, Krebs cycle; *M-TPP-T*, mitochondrial thiamine pyrophosphate transporter; *OAA*, oxaloacetate; *Succ-CoA*, succinyl-coenzyme A; *ThDP* (or *TPP*), thiamine diphosphate; *α-KG*: α-ketoglutarate. **(B)** Intracellular dotted arrows: alternative pathways for pyruvate–lactate axis and for HIF-1 during Hypoxia/pseudo-hypoxia (e.g., TD).

Intracellular-free thiamine is phosphorylated into thiamine monophosphate (ThMP), then thiamine diphosphate (ThDP) [also known as thiamine pyrophosphate (ThPP)], and finally thiamine triphosphate (ThTP). In the enterocyte, thiamine is phosphorylated to ThDP by thiamine pyrophosphokinase (TPK), before being transported from the cell apex toward the opposite pole. Most of this ThDP is then hydrolyzed to cross the basolateral membrane using an adenosine triphosphate (ATP)-dependent transporter. From here, thiamine gets into the portal blood to reach the liver and tissues via different pathways. Besides the classic hTHTRs, other carriers include alkaline/acid phosphatase transport and the organic cation transporter groups (OCT1), acting as hydrosoluble organic cations (1, 16–18, 21). Thiamine subsequently joins the systemic bloodstream either as a free form or incorporated in circulating cells, mainly erythrocytes (Figure 1A). It targets body cells that utilize glucose as their main fuel; however, thiamine tissue tropism depends on the degree of expression of key transporters on cell membranes in the major body systems (splanchnic, nervous, muscular and renal systems, and the placenta) (1, 2, 16–18, 21, 22).

In the splanchnic system, the liver and the pancreas are the two most important targets (21, 23). Recent evidence indicates that human colonic epithelial cells express specific ThDP transporters (*SCL44A4*) able to uptake thiamine, which is a local by-product of gut microbiota in infancy (Prevotella enterotype 2). It is not yet fully understood whether this microbiome-related thiamine pool is solely used *in situ* or contributes to whole body thiamine homeostasis (24, 25).

In the nervous system, central neurons – known for their high oxidative metabolism – astrocytes, and peripheral nerves are involved in the biosynthesis of a number of neurotransmitters, including acetylcholine (26, 27). Evidence suggests that the brain appears to be the ultimate target of thiamine (1, 2, 28, 29). Thiamine crosses the blood–brain barrier via carrier proteins to reach select regions of the brain, but the exact pathophysiology of this tropism is poorly understood (30–32). There are key differences in the distribution of thiamine derivatives throughout the brain, providing some insight into thiamine’s apparent preference for certain neuronal tissues. The ThDP form is mainly present in neurons and is involved in cerebral oxidative metabolism. Conversely, the ThTP form, approximately 10% of the cerebral thiamine pool, is involved in membrane excitability and nerve conduction, acting as a modulator of the permeability of sodium chloride channels (29, 30, 33, 34). Similarly, the distribution of thiamine-related enzymes is compartmentalized, especially those with rate-limiting properties, which play a role in cerebral energy utilization. Therefore TD might cause brain tissue injury by inhibiting metabolism in cerebral regions with higher metabolic demands and high thiamine turnover (34–36). The magnitude of the rate of thiamine uptake by the blood–brain barrier highlights the huge cerebral demand for thiamine and the need for its continuous supply for brain activity (22, 30, 32). *In vitro* and *in vivo* kinetics reinforce the idea that thiamine turnover is not homogeneous in the brain. It is higher in areas vulnerable to TD, and lower in other structures, such as the cerebral cortex (a relatively less sensitive region) (1, 30, 32, 34, 35). TD neurotropic effects are observed in various histological pathways of the brain, specifically causing disruption to the blood–brain barrier rich in endothelial mitochondria, choroid plexus dysfunction, differential damage in aerobic neurons and anaerobic astrocytes, microglial activation, perturbation in the glutamate system (with its specific transporters), altered dynamics of intracellular calcium and endoplasmic reticulum stress (27, 29, 31, 37–41).

Smooth, skeletal and particularly cardiac myocytes, known for their high aerobic metabolism, are all affected by TD. Their dysfunction might result in the early manifestations of TD, such as gastrointestinal paresis and heart failure (6, 42, 43). In the kidneys, the determinants of thiamine renal clearance are glomerular filtration rate (GFR) and the saturable reabsorptive capacity of proximal tubules through the thiamine/H^+^ antiport using hTHTRs, similar to polarized enterocytes (2, 18, 20, 44).

Lastly, during pregnancy, thiamine crosses the placental brush-border epithelium through a specific carrier (45, 46). This active transfer is essential for thiamine supply and may have a major impact on fetal growth and development (47).

### Intracellular Thiamine Metabolism

As previously outlined, inside the cytosolic compartment, free thiamine is first converted into the phosphorylated metabolites ThMP, ThDP, and ThTP. Adenosine forms have also been identified, e.g., adenosine thiamine triphosphate (AThTP) and diphosphate (AThDP), however their function remains unclear (33).

The synthesis of ThDP from free thiamine requires TPK, ATP, and magnesium, the deficit of which can limit clinical response to thiamine treatment (48, 49). ThDP is the biologically active form in the body (>80%) and serves as the most potent cofactor for thiamine-requiring enzymes (Figure 1B) (34). Most of the ThDP enters the mitochondrial matrix via the mitochondrial thiamine pyrophosphate transporter family (M-ThPP-T) located in its inner membrane (Figure 1C3) (50). Of note, mutation in M-ThPP-T results in Amish lethal microcephaly–neuropathy (50).

Thiamine acts as a crucial cellular cofactor for several metabolic enzymes studied in biochemical models (1, 2), namely the pyruvate dehydrogenase (PDH) multi-enzyme complex responsible for the mitochondrial decarboxylation of pyruvate to acetyl-CoA, the precursor to the Krebs cycle (Figure 1C3) (51), and alpha-ketoglutarate dehydrogenase (α-KGDH), responsible for the mitochondrial oxidative decarboxylation of α-ketoglutarate to succinyl-CoA (Figure 1C3) (36, 52). It also serves as a cofactor of cytosolic transketolase, a rate-limiting enzyme in the pentose monophosphate non-oxidative pathway leading to ribose then to nucleic acid synthesis (Figure 1C1) (53).

Upstream of the Krebs cycle, any functional defect in the PDH complex leads to anaerobic metabolism with an increase in cellular pyruvate and lactate (Figure 1B). This results in a build-up of these substrates in cerebrospinal fluid (CSF) and blood causing an elevation in the plasma anion gap (54–57). A recent report indicates that the consequent detrimental type B lactic acidosis is associated with a pseudo-hypoxia with no actual evidence of poor tissue perfusion or oxygenation. As a consequence, hypoxia-inducible transcription factor 1-alpha (HIF1-α) is increased and upregulates thiamine transporter gene expression [ThTr2 (*SLCA19A3*)] (Figures 1B,C2,C3) (58). Within the mitochondrial matrix, any earlier failed activities of PDH and α-KGDH can result in reduced ATP synthesis, which can induce an oxidative insult to the cell. Cascade reactions lead to the generation of damaging reactive oxygen species (ROS) and, ultimately, to apoptotic cell death (28, 31, 36, 59, 60). Additionally, in order for the Krebs cycle to function correctly it requires the operative integrity of PDH and α-KGDH enzymes that contribute to the synthesis of myelin and the neurotransmitter acetylcholine. Fully functional α-KGDH also preserves the physiological levels of other substrates (glutamate, gamma-amino butyric acid, and aspartate) (29, 36, 51).

Current research is investigating the role of thiamine in immune system regulation. There is evidence that its non-coenzymatic function is involved in modulation of anti-inflammatory processes and the expression of cytokine mediators (1, 3, 4).

## Conditions and Risk Factors Leading to TD in Tropical Pediatrics

Thiamine deficiency global prevalence is poorly documented. It principally affects precarious communities where children are most vulnerable and where dietary habits rely on refined processed cereals or tubers (e.g., rice, wheat, cassava), notably in Southeast Asia (5, 6, 10, 61–64), Africa (65–67) and, the Americas (68). These reports on TD prevalence vary from 13.4% of children admitted to hospital without signs of beriberi in Laos, 30% of Laotian children with uncomplicated malaria, 40% of severely malnourished children from Jamaica and Ghana, and up to 100% of children with respiratory distress and tachycardia in one Indian study.

Thiamine deficiency can result from various mechanisms that are not mutually exclusive. The most frequent mechanisms are insufficiency of dietary thiamine (enteral–parenteral supply) and poor intestinal absorptive capacity during malnutrition, tropical enteropathy, or secondary to surgical resection of large portions of the gastrointestinal tract (11, 69–71). A relative inadequacy of thiamine content to caloric ratio (high carbohydrate diets) is also common. It is this thiamine–calorie imbalance that is responsible for TD in heavy drinkers of sweet drinks (72, 73) and contributes to the onset of TD when dextrose-based fluids are administered without thiamine supplementation in critically ill patients (74).

Poor thiamine intake may be due to losses from food secondary to pre-cooking and food processing (e.g., repetitive rice washing), a restrictive diet due to cultural habits (75) and ingestion of anti-thiamine factors (tea leaves, betel nuts, and coffee) or thiaminases (e.g., fermented raw fish, mycotoxins, stored food, and larvae) that break down and, thus, inactivate thiamine (76, 77). Excessive loss of thiamine from the body, such as renal (loop diuretics, osmotic diabetic diuresis) or digestive losses (chronic diarrhea, hyperemesis), can also precipitate TD. At cellular level, two main mechanisms contribute to TD – impaired uptake or increased demand. Impaired cellular uptake of thiamine can be due to defects in thiamine transporters or specific enzymes (mutation, hypomagnesemia, drug-induced interaction), or reduced levels of ThDP. Increased cellular demand of thiamine occurs in hypermetabolic states during critical illness, e.g., severe infections, shock, burns, fever, hyperthyroidism.

Exposure to toxic substances, such as alcohol can also affect thiamine metabolism. When this occurs during pregnancy, the fetus may be indirectly affected, as observed in fetal alcohol syndrome (45–47, 78). Maternal TD associated with excessive alcohol consumption is usually secondary to inadequate thiamine intake or decreased intestinal absorption due to reduced expression of ThTr-1. In addition, impaired intracellular thiamine utilization affecting the TPK activity results in a low bioavailability of the active cellular ThDP due to ethanol and acetaldehyde exposure (2, 79–81).

In humanitarian fields, TD occurring in epidemic proportions has been described following abrupt food shortage caused by disasters, famines, conflicts, or large population displacement (66, 67, 76, 77, 82). In tropical pediatrics, the main cause of TD is poor thiamine intake, either by the child or by the mother for young infants. Associated co-morbidities are common and increase TD risk, for example, sepsis and shock during complicated severe acute malnutrition (SAM) are frequent and lead to an increase in mortality (83). Poor absorption, increased digestive losses and systemic inflammation can be aggravating cofactors in critically ill children. Additionally, pediatric intensive care practices in many resource-limited settings do not include thiamine as a component of treatment, increasing TD risk even further. In emergency and tropical contexts, awareness of the clinical spectrum of TD and recognition of such cases could have a major impact on the management of sick children and, consequently, on under-five mortality (10, 56, 61, 67, 68, 82–84).

## Syndromes of TD in Children

### Classic Forms of Beriberi in Infants

The earliest presentation of TD is in breastfeeding infants (1–3 months of age) with non-specific signs, including a “loud piercing cry” and colic (85). Refusal to breastfeed, constipation, vomiting, and agitation may also be present. Eventually edema, cyanosis, and congestive heart failure may appear. Shoshin beriberi, a fulminant form of congestive heart failure (cyanosis without edema) with type B lactic acidosis, has also been documented in infants (6, 54–56, 67, 71, 86). The administration of loop diuretics in such cases may unexpectedly worsen TD-related congestive cardiac failure due to increased renal thiamine clearance (87, 88), and TD should be suspected in any cardiogenic shock not responding to appropriate therapy (6). If undetected at this stage, death can occur within hours but prompt recognition and treatment with injectable thiamine can rapidly reverse the clinical picture and drastically improve the prognosis.

In this acute infantile form, TD results from a low thiamine content in breast milk among deficient, but mostly asymptomatic, mothers. Lactic acidosis may result in generalized symptoms, such as lethargy, irritability, anorexia, tachycardia, and tachypnea (64, 89). These clinical manifestations are probably secondary to dysfunction of mitochondrial energetics in the heart and smooth muscle (particularly the gastrointestinal tract), and an autonomic nervous system insult (6, 42, 43, 90). Due to the non-specific presentation, TD is often overlooked or misdiagnosed as typhoid fever, sepsis, malaria, pneumonia, or decompensated congenital cardiopathy in infants (5, 6, 71). TD may even be a cause of sudden infant death in this young age group (86, 90).

Later, at 4–7 months, the infant is more likely to present with an aphonic form. After increasing cough and dyspnea, the cry changes from hoarse to soundless (“aphonic cry”). Similar to the younger infant, without treatment this condition can evolve into severe acute congestive heart failure, edema, respiratory distress, and eventually death within a few days (86). The clinical appearance at this stage is easily mistaken for severe croup, though TD is usually an afebrile condition. Laryngeal symptoms in TD are due to a Xth cranial nerve palsy caused by local nerve damage and voice changes may be worsened by laryngeal edema and generalized edema secondary to congestive heart failure, which are both observed in TD (6, 67, 89, 91).

### Pediatric Neurological Manifestations of TD

Thiamine deficiency can present with a broad range of neurological signs in children, such as anorexia, irritability, agitation, muscle pain, diminished or abolished deep tendon reflexes, ataxia, paralysis, and a progressively altered level of consciousness (67, 86, 92). Given the varied clinical presentations, any unexplained severe neurological signs or symptoms should raise the suspicion of TD. Nevertheless, three main neurological syndromes are described in children: pseudomeningitic beriberi in the first year of life, Enrick’s encephalopathy, and impaired cognitive development associated with mild chronic TD throughout infancy.

The onset of the pseudomeningitic form of infantile beriberi occurs at around 6–12 months of age. Clinical manifestations include muscular fasciculation, nystagmus, ophthalmoplegia, tense fontanel, seizures, and coma. If a lumbar puncture is performed, CSF is found to be clear, with slightly high lactate and low 5-hydroxyindoleacetic acid (5-HIAA) levels (22, 86, 93, 94). This form of TD can be easily confused with encephalitis, meningitis, cerebral-malaria, and vitamin A intoxication and should be included in the differential diagnosis of meningism in children (91, 95).

Nystagmus and ophthalmoplegia are attributed to lesions of the pons vestibular nuclei, and the nuclei of the third and the sixth cranial nerves of the periaqueductal region, respectively. The absence of significant destruction of neurons in these nuclei might explain the rapid reversal of the ocular and vestibular disturbances following thiamine administration. This suggests a continuous evolution from the “biochemical stage” to the irreversible organic lesions that are seen if left untreated (36, 52).

Although classically associated with alcoholism, Wernicke syndrome as a manifestation of TD is not limited to adults. Some children may develop a truncated Wernicke-like syndrome without ataxia, while the classic triad of ophthalmoplegia or nystagmus, ataxia and altered consciousness, is more common in older children (69, 96–100). Typical lesions of TD should be sought in the cerebral targets of thiamine: the cerebellar–pons axis, the midbrain (periaqueductal gray matter and tectum), the mammillary bodies, and the adjacent hypothalamic and thalamic regions (97, 98). Unlike in adults, pediatric brain MRI of Wernicke-like syndrome displays particular lesions in the frontal lobe and basal ganglia, notably the striatum and putamen. However, both adults and children affected by TD show the same symmetrical high-intensity signal on T2 weighting in mammillary bodies and periaqueductal and thalamic areas (97, 98, 100). Cerebral Spectrum MR displays a significant lactate doublet spike with changes in N-acetylaspartate/creatine and choline/creatine ratios, markers of neuronal damage and disruption to myelination, respectively (97, 100). Although rarely accessible in resource-limited settings, early detection of these lesions on neuroimaging could significantly improve clinical outcome (97, 98, 100).

In addition to the acute neurological presentations described, more subtle neurological impairments due to chronic TD in infancy have been identified, particularly affecting cognitive development. Animal studies and clinical observations suggest that an undetected impairment in neuropsychological development occurs during mild chronic thiamine depletion. In the long term, there is neurological damage causing cognitive and psychomotor troubles, alteration in the syntactic and lexical modalities of language acquisition, and seizures causing neurological sequelae (94, 101, 102). This highlights the importance of early diagnosis of TD during this critical period in infancy of accelerated learning and development of essential cognitive skills.

### Sepsis and Severe Acute Conditions Associated With, or Exacerbated by Thiamine Deficiency

Severe acute conditions are often associated with functional or true TD in adults (103). Lima et al. (104) found that prevalence of TD on hospital admission was 28% in a Brazilian pediatric intensive care unit,(104) while Khounnorath et al. (5) documented biochemical TD in up to 13.4% of sick infants without evidence of overt beriberi (5). In tropical pediatrics, severe infections often occur on the background of malnutrition, with significant macro and micronutrient deficiencies already present. This may explain the much worse prognosis of septic shock in children with SAM, who potentially have an underlying TD exacerbated by sepsis (83). TD has been linked to an increase in mortality in severe acute conditions and shock, associated with both type A and B lactic acidosis (5, 57, 103). However, although Costa et al. (105) confirmed the high prevalence of TD in septic shock in intensive care patients (up to 71%), they were unable to show a consistent correlation with increased mortality (105).

Complex metabolic conditions may indirectly lead to TD or functional TD, for example, through increased digestive losses, poor thiamine intake during severe illnesses, and high turnover associated with hypermetabolic states, which leads to exhaustion of thiamine stores (74, 106). Critically ill children are often fasting for the first 24 to 48 h of admission and parenteral nutrition in resource-poor settings is lacking. The use of dextrose-based maintenance fluids further potentiates TD risk due to the high consumption of thiamine in the glucose metabolic pathway, as previously outlined.

In sepsis, shock syndromes and severe acute conditions, the current understanding is that hyperlactatemia (a prognostic indicator) is secondary to both type A acidosis, related to hypoxia, and type B acidosis, related caused by dysfunction of PDH in TD. Severe acute conditions lead to endogenous hypermetabolism with mitochondrial respiratory dysfunction. This causes an increase in both oxygen and thiamine consumption (as a PDH cofactor) because of a higher metabolic demand. The resulting functional TD induces a pseudohypoxic state, with the accumulation of pyruvate and lactate, which in turn trigger the release of HIF1-α. HIF1-α, bound to its β subunit, regulates the expression of many genes, notably those involved in metabolic adaptability to hypoxia (gene expression for angiogenesis and erythropoietin synthesis) and innate system modulation (Figure 1C2) (58, 107). HIF1-α also favors anaerobic pathways via LDH gene expression and downregulation of the PDH complex (through PDK activation) in the mitochondria (Figure 1C3). In addition, HIF1-α is known to upregulate both Glut (glucose transporter) to increase uptake of glucose into the cell to meet the immediate demand for ATP via anaerobic pathways, and ThTr2 (thiamine transporter) synthesis at the transcriptional level in order to increase the intracellular thiamine pool pending the recovery of mitochondrial function (Figure 1C2).

This adaptive HIF1-α response may be beneficial in the face of rapid depletion of the thiamine pool caused by excessive utilization in severe acute conditions. In other words, the HIF1-α system creates the optimal cellular conditions to restart aerobic respiration once oxygenation and thiamine supply are restored. This is consistent with Donnino et al.’s (108) recent findings that show the significant positive effect of administration of high-dose thiamine (400 mg) in a subgroup of adults with septic shock and TD (108). Moreover, the Zhu study (109) suggests that sepsis *per se* might differentially block thiamine-carrier protein synthesis at the mRNA level and impair mitochondrial energetics in rat diaphragms. This would limit cellular thiamine entry, thus, another precipitating factor for functional TD (109). In these circumstances, much higher thiamine dosage than that recommended for beriberi would be required to counteract this phenomenon, as observed in Donnino’s trial (108).

The physician should suspect underlying TD in critically ill children with persistent lactic acidosis or increased plasma anion gap, as well as in any condition resulting in a hypermetabolic state such as shock, sepsis, large burns, poly-trauma, unexplained congestive heart failure, congenital heart disease, diabetic ketoacidosis, and severe malaria (5, 54, 63, 71, 110–114).

### Induced-Thiamine Deficiency in Refeeding Syndrome

Thiamine deficiency or subclinical low vitamin B1 levels may be present in children with SAM, as reported in Jamaican and Ghanaian studies (~40–43%) (65, 68). This is most likely the result of a poor and monotonous diet, associated with malabsorption and thiamine loss caused by malnutrition-related diarrhea as well as a degree of intestinal epithelial atrophy (69, 84).

Refeeding syndrome is a potentially fatal complication of SAM management, especially when the introduction of food is too fast. Rapid initiation of nutritional rehabilitation also increases intracellular thiamine turnover which, on a background of pre-existing low whole body thiamine status, can precipitate the onset of true TD and may contribute to the mortality linked with refeeding syndrome (115–117).

Despite overlapping clinical pictures, refeeding syndrome and TD associated with refeeding do not share the same pathogenesis, with TD being a consequence of refeeding rather than a part of the syndrome (117). Refeeding syndrome itself is characterized by electrolyte imbalances caused by intracellular shift (e.g., hypophosphatemia, hypokalemia, hypomagnesemia), and leads to congestive heart failure with edema, neurological, and hematological complications. In induced TD, refeeding stimulates insulin production leading to protein synthesis and increased thiamine demand due to glucose utilization. Pre-existing low thiamine levels in SAM are further reduced by this increased cellular consumption of thiamine. As a result, mitochondrial enzyme complexes (PDH and alpha-KGDH, dependent on their cofactor level) become dysfunctional and the glucose pathway is blocked at the level of pyruvate. The only alternative is for pyruvate to be channeled through the anaerobic pathway leading to type B lactic acidosis, a hallmark of acute TD.

Signs of refeeding syndrome and induced TD are often overlooked or misinterpreted as sepsis, cardiac failure, pneumonia, or sudden death (116). In addition, thiamine assays are usually processed in highly specialized laboratory facilities, therefore, are rarely available in settings where SAM commonly occurs, making confirmed diagnosis rare in resource-limited settings (115). For this reason, diagnosis of TD in this context relies on a high level of clinical awareness and suspicion.

Refeeding syndrome requires careful management, including adjustment of therapeutic milk volumes with consideration of calorie load, gradual correction of electrolyte imbalances and administration of a thiamine loading dose to prevent or correct refeeding-induced TD. Sydney Children’s Hospital Guidelines and Cape Town Pediatric Interest Group suggest 2 mg/kg of thiamine daily during the first week of SAM treatment to prevent refeeding syndrome and its consequences (118, 119). Although current international therapeutic feeding guidelines are designed to provide optimal SAM management and avoid complications, high-dose thiamine at initiation of treatment is not the current practice in humanitarian settings (84). Since we know that a number of children with complicated SAM have borderline thiamine stores, even very cautious introduction of feeds may trigger refeeding syndrome and induced TD without the addition thiamine supplementation.

### Acute Clinical Syndromes Associated with Thiaminases

Traditional diets, including fermented fish (containing thiaminase) and betel nut (containing thiamine antagonists) can precipitate symptoms of TD in a nutritional context of high carbohydrate monotonous diet, such as polished rice (66, 77).

However, the most striking clinical manifestation of TD associated with thiaminases is African Seasonal Ataxia. Small case series’ or large outbreaks (described in western Nigeria), related to the consumption of thiaminase-containing *Anaphe venata* larva, are described during the rainy season (91, 120). Patients develop nausea, vomiting, and dizziness within hours of thiaminase ingestion. Subsequently, fulminant neurological signs develop with nystagmus, tremor, cerebellar ataxia, dysarthria, confusion, and eventually coma. If the diagnosis is suspected early, treatment with thiamine bolus rapidly relieves symptoms and reverses coma within 72 h, suggesting a massive depletion of whole body thiamine caused by thiaminase.

A recent report of infant botulism was found to be associated with clostridium production of thiaminase I, inducing TD. It was speculated that the paralysis of this patient due to clostridium neurotoxins was in fact aggravated by TD. Indeed, the administration of thiamine resulted in faster improvement and a shorter clinical course (121).

## Diagnosis and Treatment

Thiamine deficiency can be investigated using plasma, erythrocytes, whole blood ThPP levels (22, 33), and urinary excretion of thiamine before and after exogenous thiamine administration (7). However, serum or whole blood thiamine has poor sensitivity and specificity in severe acute conditions as it decreases during systemic inflammation, and represents only a small part of the whole body thiamine pool. Erythrocyte transketolase activity more accurately evaluates the thiamine status of the body and is, therefore, used as standard (9). MR imaging showing specific lesions can also be very helpful in early detection of neurologic features of TD (97, 100). However, none of these investigations allow immediate diagnosis of TD in life-threatening conditions and they are rarely available in resource-limited settings.

Consequently, the diagnosis of TD in such contexts is a real challenge, especially considering its wide and non-specific clinical spectrum and potentially fatal prognosis. A high level of clinical suspicion should be demonstrated in the following situations (5, 6, 54): suspicion of infantile beriberi; unexplained neurological signs, encephalitis, and cardiac failure; early clinical deterioration after initiation of feeds in malnutrition; sepsis (including in SAM); severe burns; major trauma; hypoxia; and unresponsive lactic acidosis. Rapid point of care lactate testing can be used to confirm persistent lactic acidosis despite correction of shock, and could help to raise the suspicion of TD (54, 57, 58). However, in the absence of specific diagnostic tests, the only way to diagnose TD is to carry out a therapeutic thiamine challenge. Considering its safety profile and wide dosage range, in such cases, thiamine can be administered by slow intravenous injection over 30 min. In severe acute conditions caused by TD, rapid clinical improvement will be seen (within hours or days) following thiamine administration.

Evidence-based pediatric thiamine dosage recommendations for severe acute illness are lacking. Doses found in this literature review vary from 50 to 1500 mg depending on the clinical condition (7, 9, 86, 91, 122), with neurological presentations potentially requiring higher doses and having a longer recovery time (a few days). A recent study showed that thiamine-deficient adults with septic shock in ICU had a significantly lower mortality if they received thiamine 200 mg twice daily for a week compared to placebo (108). Rao and Chandak (6) used 150 mg IV thiamine to treat breast-fed infants under 6 months of age presenting with cardiac failure and/or tachypnea (6), while Qureshi et al. successfully treated lactic acidosis in younger infants (aged 32 days to 4 months) with 100 mg of thiamine daily (89). In both studies, thiamine was well tolerated.

Given this evidence, early thiamine injection in children with severe acute conditions should be considered as a complementary resuscitation tool, especially when there is co-existing malnutrition or where dextrose-based fluid is used to resuscitate (6, 90, 99). Switching to the enteral route should be considered as soon as possible after the initial thiamine bolus (54, 123, 124). In addition to optimal dosage, practical aspects of drug dilution and calculation must be taken into account in resource-poor settings where staff are not always highly qualified. For example, 100 mg is an easy dose to prepare and administer for preparations of 100 mg/ml of thiamine.

According to the literature, prevention of TD during the early refeeding phase in complicated SAM initially requires 10–30 mg of thiamine daily followed by 5–10 mg daily for a month (74, 119, 125). This is not standard practice in humanitarian programs and would provide 10–20 times more than currently recommended in therapeutic protocols, as shown in Table 1 (84, 119). One sachet of ready-to-use therapeutic food (RUTF) or 600 ml of therapeutic milk [either of F-75 (75 kcal/100 ml) or F-100 (100 kcal/100 ml)] contain an average of 0.5 mg of thiamine (84); thus, the average daily intake represents 1–2 mg at most. This is even more significant in infants under 6 months of age with SAM who are at greater risk of developing TD. These babies receive neither RUTF, F-75 nor F-100 but only breast milk or specially diluted F-100, and breastfeeding mothers are rarely supplemented. Infants under 6 months should, therefore, be supplemented with thiamine (2 mg/kg) in order to minimize the risk of precipitating TD.

**Table 1:** **Thiamine content of therapeutic milk and estimated thiamine needs in children during the acute phase of refeeding in SAM**.

**Body weight (kg)**	**Approximate amount of F-75 (milliliter and equivalent of thiamine content) given in 8 meals/day in acute stabilization phase according to refeeding protocols [WHO (84)]**	**South African and Australian guidelines for prevention of refeeding syndrome (2 mg/kg of thiamine) (118, 119)**
5	8 × 85 ml/day (~0.5 mg of thiamine)	10 mg of thiamine
7	8 × 120 ml/day (~0.8 mg of thiamine)	14 mg of thiamine
10	8 × 170 ml/day (~1.1 mg of thiamine)	20 mg of thiamine
15	8 × 250 ml/day (~1.7 mg of thiamine)	30 mg of thiamine

*SAM, severe acute malnutrition; F-75 is the therapeutic milk used during early refeeding (75 kcal/100 ml). As an example, a 7 kg child with complicated SAM would be given eight meals of 120 ml of F-75 on admission. Applying above guidelines (third column) would represent approximately 17 times more thiamine*.

Adding weight to the benefits of thiamine supplementation, Luxemburger et al. (82) showed thiamine distribution in a population with high prevalence of TD (Karen refugees’ camps) to be clinically significant. Pregnant women with overt signs of TD received 100 mg of thiamine daily until delivery, followed by 10 mg weekly until 9 months after birth for both mother and infant. Highly relevant results showed overall reductions in infant mortality rate from 183 to 78 per 1000 live births, case fatality of infantile beriberi from 100 to 7%, postnatal deaths by 79% (CI_95%_ 65–87%), and mortality attributable to beriberi from 73 to 5 per 1000 (*P* < 0.0001) (82).

In summary, based on current evidence, we propose that a therapeutic challenge of 100 mg of thiamine should be given in acute severe illness and in clinically evident TD. For prevention of TD in high-risk situations, such as nutritional and humanitarian crises, refugee, and displaced populations, mass thiamine weekly distribution of 10–30 mg could be considered. The thiamine content of therapeutic foods should also be re-evaluated to minimize TD during the initial phase of refeeding. This must include RUTF, as most SAM patients are treated as out-patients. Finally, pregnant women with a suspicion of TD should receive thiamine treatment during pregnancy, and both mother and baby should continue supplementation while breastfeeding.

## Conclusion

This review highlights new perspectives on pathophysiological and clinical correlations in pediatric TD. Thiamine is an intracellular micronutrient with ubiquitous body distribution and plays a fundamental role in mitochondrial energetics within its key target organs. The article outlines the intracellular thiamine transporter traffic and explains the association of TD with type B lactic acidosis and dysfunction of the Krebs cycle, indicating that TD should be considered as a form of acquired functional mitochondrial disease in pediatrics. This review also sheds light on the important role of thiamine in refeeding syndrome and in critical illness. In such circumstances, there is evidence of a mismatch between the increased endogenous metabolic requirements and cellular thiamine availability.

Thiamine deficiency has a large clinical spectrum, and as such it is frequently misdiagnosed, sometimes with fatal consequences or permanent neurological sequelae. Even classic beriberi is still often unrecognized, and increased clinical awareness is essential. Early treatment with thiamine has the potential to rapidly reverse clinical signs and minimize sequelae, before the onset of fixed lesions. The lack of diagnostic capacity in resource-poor settings justifies the use of a therapeutic thiamine challenge in cases with high clinical suspicion. It is an effective, inexpensive, and easy to administer medication.

Many risk factors for TD coexist in tropical pediatrics, making it a highly relevant public health issue. This review provides an opportunity to raise awareness of TD prevalence in malnourished and critically ill children, and to consider early systematic thiamine treatment and clarification of the role of thiamine in the algorithms of metabolic resuscitation. Finally, it calls into question the current thiamine content of RUTF and milk in therapeutic feeding programs, and provides arguments for added thiamine supplementation in therapeutic foods for malnutrition.

## Author Contributions

LH is in charge of clinical and therapeutic aspects. BR is in charge of physiological and some clinical aspects. NL is in charge of critical review, relevance, medical, and English editing. DMG is in charge of references and critical medicine aspects. All authors contributed to the coherence of the final draft. All authors collectively agreed with the scientific content of this article.

## Conflict of Interest Statement

Authors declare having no conflict of interest and received no specific funding for this study.

## Abbreviations

AThDP, adenosine thiamine diphosphate; AThTP, adenosine thiamine triphosphate; hTHTRs, human thiamine transporters; M-ThPP-T, mitochondrial thiamine pyrophosphate transporter; Spectrum MR, spectrum magnetic resonance; TD, thiamine deficiency; ThDP, thiamine diphosphate; ThTP, thiamine triphosphate; ThTr1, thiamine transporter 1; ThTr2, thiamine transporter 2; TPK, thiamine pyrophosphokinase.
